# Treatment of a Critically Ill COVID-19 Patient with the Seraph 100 Microbind Affinity Filter

**DOI:** 10.1055/s-0041-1727121

**Published:** 2021-04-14

**Authors:** Anke Pape, Jan T. Kielstein, Tillman Krüger, Thomas Fühner, Reinhard Brunkhorst

**Affiliations:** 1 Kuratorium für Dialyse und Nierentransplantation, Pelikanplatz, Hannover, Germany; 2 Department of Nephrology, Angiology and Rheumatology, KRH Klinikum Siloah, Hannover, Germany; 3 Department of Nephrology, University Hospital of Essen, University of Duisburg-Essen, Essen, Germany; 4 Medical Clinic V Nephrology, Rheumatology, Blood Purification, Academic Teaching Hospital Braunschweig, Braunschweig, Germany; 5 Department of Respiratory and Critical Care Medicine, KRH Klinikum Siloah, Hannover, Germany

**Keywords:** SARS-CoV-2, extracorporeal treatment, circuit failure, D-dimer

## Abstract

The coronavirus disease 2019 (COVID-19) pandemic has a serious impact on health and economics worldwide. Even though the majority of patients present with moderate and mild symptoms, yet a considerable portion of patients need to be treated in the intensive care unit. Aside from dexamethasone, there is no established pharmacological therapy. Moreover, some of the currently tested drugs are contraindicated for special patient populations like remdesivir for patients with severely impaired renal function. On this background, several extracorporeal treatments are currently explored concerning their potential to improve the clinical course and outcome of critically ill patients with COVID-19. Here, we report the use of the Seraph 100 Microbind Affinity filter, which is licensed in the European Union for the removal of pathogens. Authorization for emergency use in patients with COVID-19 admitted to the intensive care unit with confirmed or imminent respiratory failure was granted by the U.S. Food and Drug Administration on April 17, 2020.

A 53-year-old Caucasian male with a severe COVID-19 infection was treated with a Seraph Microbind Affinity filter hemoperfusion after clinical deterioration and commencement of mechanical ventilation. The 70-minute treatment at a blood flow of 200 mL/minute was well tolerated, and the patient was hemodynamically stable. The hemoperfusion reduced D-dimers dramatically.

This case report suggests that the use of Seraph 100 Microbind Affinity filter hemoperfusion might have positive effects on the clinical course of critically ill patients with COVID-19. However, future prospective collection of data ideally in randomized trials will have to confirm whether the use of Seraph 100 Microbind Affinity filter hemoperfusion is an option of the treatment for COVID-19.

## Introduction

About 102 years after the Spanish flu that took approximately 50 million lives, the novel coronavirus (2019-nCoV), also known as severe acute respiratory syndrome coronavirus 2 (SARS-CoV-2), leads to coronavirus disease 2019 (COVID-19) and is considered unprecedented in its effect on global health and economy. Notwithstanding drastic measures to contain the virus, its spread continues and the death toll rises. Even though an enormous progress in clinical medicine has been made over the last century, we have currently no efficient therapy in our toolbelt to treat COVID-19 except for dexamethasone. 1 Given the urgency of a public-health emergency, which has been declared a pandemic by the World Health Organization, many therapeutic options above and beyond drug therapy are currently explored. So far the extracorporeal strategies that have been discussed and used in COVID-19 are mainly aimed to reduce the cytokine storm. 2 The Seraph 100 Microbind Affinity filter has recently been introduced for the elimination of bacteria 3 and other pathogens from the blood. 4 Authorization for emergency use in patients with COVID-19 admitted to the intensive care unit (ICU) with confirmed or imminent respiratory failure was granted by the U.S. Food and Drug Administration on April 17, 2020. The rationale for the approval was the fact that viral RNAemia is frequently (up to 78%) seen in critically ill patients where it is related to the severity of the disease. 5 Moreover, the monomeric and trimeric SARS-CoV-2 spike glycoprotein binds tightly to immobilized heparin, 6 the functional backbone of the Seraph 100.

Here, we describe the successful treatment of a critically ill COVID-19 patient with the Seraph 100 Microbind affinity filter. The markedly elevated lactate dehydrogenase (LDH), N-Terminal propeptide of brain natriuretic peptide (NT-proBNP), and D-dimer fell during the treatment. In total, 3 days after treatment with Seraph 100, the patient could be extubated and left the ICU after just 9 days of treatment.

## Case Report

In early April 2020, a 53-year-old Caucasian firefighter presented to the emergency department of our tertiary care hospital with fever (temperature 40.5°C). Main symptoms were nausea, vomiting, and diarrhea accompanied by dry cough, headache, and muscle pain for 7 days. Shortness of breath was denied. He had returned from skiing in Brixen, Southern Tyrol, Italy 7 days prior to hospital admission. Five people in his skiing group had been tested positive for SARS CoV-2. Besides a reflux esophagitis years ago, the patient has no significant medical history.

The general condition of the febrile male was significantly reduced. The pulmonary examination remained unremarkable. Vital signs showed a blood pressure of 129/84 mmHg, a heart rate of 75 bpm and a temperature of 40.5°C. Peripheral oxygen saturation under room air was 98%. Respiratory rate was 16/minute. The capillary blood gas analysis showed a respiratory alkalosis due to hyperventilation (pH 7.599, pCO _2_ 21.1 mmHg, pO _2_ 71.2 mmHg, base excess (BE) 0.8 mmol/L). Laboratory evaluation on admission is summarized in Table 1 . A decreased transparency in left lower lung field was seen in the chest X-ray.

**Table 1:** Course of Vital signs and laboratory data

Vital signs	Admission to the hospital	Transfer to the ICU	Intubation	Start of Seraph 100	After Seraph 100	2 days after Seraph 100	
Blood pressure (mmHg)	129/94	165/73	140/60	122/70	112/70		
Heart rate (bpm)	75	67	80	62	62		
Body temperature (°C)	40.5	38.7	38.0	37.7	38.5		
Respiratory rate (/minute)	15	20	30	BiPAP	BiPAP	cPAP/BiPAP	
O _2_ saturation (%)	98	88	98	94	94		
FiO _2_ (%)				35	35	21	
Laboratory data							Normal range
Hemoglobin (g/dL)	16.1	12.7	12.0		11.8	11.9	13.7–17.5
Leukocytes (Tds/µL)	4.82	5.48	6.16		8.01	7.97	4.24–9.07
Lymphocytes (Tds/µL)	1.54	0.53	0.89		1.85	1.80	1.32–3.57
CRP (mg/L)	47.5	234	256		243	172	<5
LDH (U/L)	380	745			505	467	135–225
D-dimer (mg/L)	Not done	3.39		15.8	2.34	1.63	<0.5
NT-Pro BNP (pg/mL)	Not done	306		517	189	48	<125

Abbreviations: BiPAP, bilevel positive airway pressure; cPAP, continuous positive airway pressure; CRP, C-reactive protein; LDH, lactate dehydrogenase; NT-Pro BNP, N-terminal propeptide of brain natriuretic peptide.

Based on the travel history, the throat swab, and the chest X-ray, the diagnosis of a viral pneumonia with COVID-19 was made. The patient was isolated on the pulmonary ward. He initially received symptomatic treatment with IV fluids, antiemetic (granisetron IV) and antipyretic (metamizole IV) therapy. About 3 days after admission, the patient developed lymphopenia (0.96 × 10 ^3^ /µL), and the CRP increased to 191.1 mg/L. At this point, azithromycine and supplemental oxygen via nasal cannulas were started. Due to respiratory deterioration, the patient was transferred to the ICU and hydroxychloroquin was started with 200 mg b.i.d. on the 5th day after admission. Respiratory exhaustion with increasing respiratory rate and beginning of desaturation occurred 24 hours after admission to the ICU requiring intubation. At the beginning of the COVID pandemic, invasive ventilation was preferred over noninvasive ventilation as the fear of virus-containing aerosols prevailed over the assumed benefit of noninvasive ventilation. An echocardiography did not show signs of pulmonary embolism or right heart failure. Due to the lack of a specific pharmacological therapy, an extracorporeal treatment using the Seraph 100 Microbind Affinity Blood Filter was established as a rescue therapy. The rationale for this approach was the fact that viral RNAemia was already seen reported in severly ill COVID-19 patients, which according to recent data account for up to 78% of all patients in the intensive care unit. 5 The decrease of viral RNA or viremia by the Seraph 100 seemed not far fetched as the coronaviruses, and especially SARS-CoV-2 with its spike glycoprotein has been shown to bind exquisitely well to immobilized heparin, 6 the functional backbone of the Seraph 100.

We informed the patient about the possibility of treatment with Seraph 100, and he consented to this treatment prior to his intubation. It is a single use extracorporeal broad-spectrum sorbent hemoperfusion device for the reduction of pathogens from the bloodstream. 4 Vascular access was obtained via double lumen catheter in the right femoral vein. The Seraph 100 was used as hemoperfusion, that is, there was no concomitant renal replacement therapy. Using an Octo-Nova (DIAMED Medizintechnik GmbH, Cologne, Germany), a blood flow of 200 mL/minute was established. After a bolus of 2,500 IE unfractionated heparin, the continuous anticoagulation consisted of 2,000 IE unfractionated heparin per hour. Treatment was well tolerated and the mean arterial pressure with inotropic support (noradrenalin 0.06 µg/kg/min or 3.33 µg/kg/h) was maintained between 122/70 and 112/70 mmHg. Oxygen saturation during the treatment was 94%, while the respirator settings remained unchanged in bilevel positive airway pressure (BiPAP) mode with an FiO _2_ of 35% and a positive end-expiratory pressure of 7 mbar and inspiratory pressure of 24 mbar, respiratory rate of 15/min.

After initiation of the therapy patient who was under propofol sedation, became more agitated, so that the dose was increased from 120 to 200 mg/h within the first 30 minutes of therapy. Additional sedation was changed to midazolam. After 70 minutes of treatment, venous return pressure of the hemoperfusion device increased and the filter clotted before the blood could be given back to the patient. The inotropic support could be stopped at this time. There were no acute changes in ventilation parameters or oxygenation. After the hemoperfusion with Seraph 100, the patients previously rapidly deteriorating clinical status stabilized so that no further intensification of ICU care was necessary. Over the next 3 days, he was weaned off the ventilator. The elevated LDH, NT-proBNP, and D-dimer levels fell. About 3 days later, the patient could be extubated and left the ICU after a total stay of 9 days.

## Discussion

Apheresis can be considered in a variety of clinical circumstances including viral infections and the overwhelming response to them. 7 To our knowledge, this is the first case of hemoperfusion with the Seraph 100 Microbind Affinity in a critically ill patient COVID- 19 in Europe. A report from two patients in the United States had been published. 8 What is the rationale to use such a device patient with severe pulmonary SARS-CoV-2 infection?

The Seraph 100 filter has been licensed in the European Union in 2019 for the removal of pathogens from the blood. The functional basis of the device are ultra-high molecular weight polyethylene beads with end point-attached heparin. Bacteria, viruses, fungi, and toxins have been shown to bind to the immobilized heparin in a similar way to the interaction with heparan sulfate on the cell surface. 4 Due to this biomimetic action, pathogens binds irreversibly to the heparin on the polyethylene beads and are thereby removed from the bloodstream.

Heparin binding is a frequent feature in viruses as this ability is important to bind heparan sulfate proteoglycans on the surface of host cells – a precondition to enter the cells through internalization. For SARS-CoV-2, it has been shown that it not only binds to heparin but also that ACE2-mediated coronavirus entry can be mitigated by heparin, a heparan sulfate-related glycan, or by genetic ablation of biosynthetic enzymes for the cell surface heparan sulfate proteoglycans. 9

Although viremia is demonstrated in a small percentage of patients, 8% in one case series, 10 detectable SARS-CoV-2 viral RNA in the blood has been shown to be a strong indicator for the clinical course. 11 Indeed SARS-CoV-2 RNA in serum at hospital admission indicates a high risk of progression to critical disease and death. 12 Moreover, patients with severe COVID-19 tend to have a high-viral load and a long virus-shedding period. 13 Platelets can be hyperactivated in association with SARS-CoV-2 RNA and thus presumably contribute to trigger the hypercoagulation and thrombosis, 14 which is however a multifaceted process that involves several pathways. 15

We suggest that D-dimers could be a surrogate parameter of ongoing and aggravating thromboembolism. We made the observation that D-dimers levels increase simultaneously at clinically deterioration and decrease with improvement. But further investigation is needed to clarify this assumption. Another mechanism that might be beneficial is the reduction of proinflammatory cytokines that had been shown for the Seraph 100 in vitro, 16 highlighting further potential therapeutic benefit.

Of note, there were two clinical findings during the Seraph 100 treatment. The first one was the agitation of the patient potentially aggravated through the removal of the sedating agents. Indeed, so far the effect of the S Seraph 100 Microbind Affinity treatment had only been investigated for anti-infective agents 17 as well as for chloroquine and hydroxychloroquine. 18 An effect on hypnotics and sedatives has not been evaluated.

The second clinical finding of interest was the rapid circuit failure due to clotting of the Seraph 100 that is packed with immobilized heparin. We know that COVID-19 patients exhibit a deranged coagulation function that might explain this finding. 19 One report from the United States in which the use of the Seraph 100 Microbind affinity filter was also reported clotting (of the vascular access) that resulted in the premature end of the treatment after 3.5 hours. 8 In contrast to this publication, we did not see a drop on body temperature.

D-dimer level has been repeatedly shown to be associated with poor outcome in COVID-19 patients. 20 As the normal half-life of D-dimers is approximately 5 hours; hence, the dramatic reduction in D-dimer levels during the Seraph treatment has to be attributed to their removal that might have caused the filter clotting. We can only speculate about the effect of the immobilized heparin on the coagulation problems in our patient. Interestingly, a recent study suggested that anticoagulant therapy seems to be associated with an improved outcome in severe COVID-19 patients. 21 The reduction of NT-ProBNP by approximately 64% can be in part attributed to the normal decay of this marker with a half-life of 120 minutes. 22 The potential effect of the Seraph 100 on NT-ProBNP could not be established as pre- and post-Seraph 100 blood samples drawn at the same to calculate the actual device clearance had not been obtained. As the clinical effectiveness of the Seraph 100 Microbind affinity filter in critically ill patients cannot be evaluated based on anecdotal reports, an online registry has been recently established (Registry for the Evaluation of Safety and Effectiveness of the Seraph 100 Microbind Affinity Blood Filter in the Therapy of COVID-19 Patients (COSA) ClinicalTrials.gov Identifier: NCT04361500). We noticed in subsequent treatments with Seraph 100 Microbind Affinity Filter a significant decrease in D-dimers without clotting of the circuit. It can be speculated that the use of propofol contributed to the clotting of the circuit. Taking midazolam for sedation and without increasing the amount of heparin, we did not see any clotting of the Seraph 100.

The intention of this case report is to show that hemoperfusion with the Seraph 100 in patients with COVID-19 is feasible, and the device is easy to handle by using standard dialysis equipment and requires no laborious preparation other than rinsing with normal saline. We would like to encourage other centers to participate in the online register COSA to obtain reliable data on effectiveness of Seraph 100 in critically ill COVID-19 patients with the goal to gain more evidence on its use in this disease. In future, randomized controlled trials with Seraph 100 will have to provide the scientific basis for the evaluation of its effect on hard clinical endpoints in COVID-19 patients.
